# Intravascular Papillary Endothelial Hyperplasia Involving the Hand: A Case Report of a Rare Entity

**DOI:** 10.7759/cureus.50902

**Published:** 2023-12-21

**Authors:** Abeer Albazali, Alsadat Mosbeh, Yashpal Manchanda, Adnan Ahmad, Fatmah Altaweel, Yasmeen Mandani, Ashok K Sharma

**Affiliations:** 1 Department of Dermatology, Farwaniya Hospital, Ministry of Health, Kuwait, KWT; 2 Department of Dermatology/Dermatopathology, Faculty of Medicine, Al-Azhar University, Cairo, EGY

**Keywords:** thumb lesion, excision, intravascular papillary endothelial hyperplasia (ipeh), subcutaneous nodule of hand, masson’s tumor

## Abstract

Masson’s tumor, also named intravascular papillary endothelial hyperplasia (IPEH), is a rare, benign vascular tumor. Evaluation by clinical features can be confused with other soft tissue tumors. Therefore, the diagnosis should be confirmed by histopathological examination.

The patient reported here is 67 years old and came to us with a small painful lesion over the left thumb of about two months duration. Histopathological examination was consistent with Masson’s tumor (IPEH) following excisional biopsy, with good functional outcomes.

To the best of our knowledge, this is the first report of this entity from Kuwait. Dermatologists and surgeons should know about this rare entity and its unusual presentation, to be able to distinguish it from similar presenting serious conditions, especially angiosarcoma. Through this report, we purport to facilitate recognition of this condition apart from some other conditions it may mimic.

## Introduction

Masson’s tumor, a rare benign neoplasm, also known as intravascular papillary endothelial hyperplasia (IPEH), can present over any part of the body, however, usually the head, neck, and upper extremities are involved [1]. This uncommon condition was first described in 1923 by Pierre Masson [2]. Although widely accepted to have no pathognomonic features [3], it usually presents as a small red or blue slow-growing nodule [4]. Today, it is not considered a true tumor but is thought to be a reactive hyperplastic vascular endothelium growth [5-7]. Surgical excision is the desirable method of treatment [1,8]. To the best of our knowledge, this is the first report of this rare entity from Kuwait.

## Case presentation

A 67-year-old Kuwaiti female, a known case of gastroesophageal reflux disease (GERD), irritable bowel syndrome (IBS), and osteoporosis, presented to our dermatology outpatient department complaining of a single small painful bluish lump that appeared on the left thumb two months ago. The lump was limited to the palmar aspect of the left thumb. There were no changes in the surrounding skin noted by the patient. Additionally, the patient denied any itchiness, bleeding, or discharge from the lump. The lesion was causing the patient discomfort and affecting her daily life activities.

**Figure 1:**
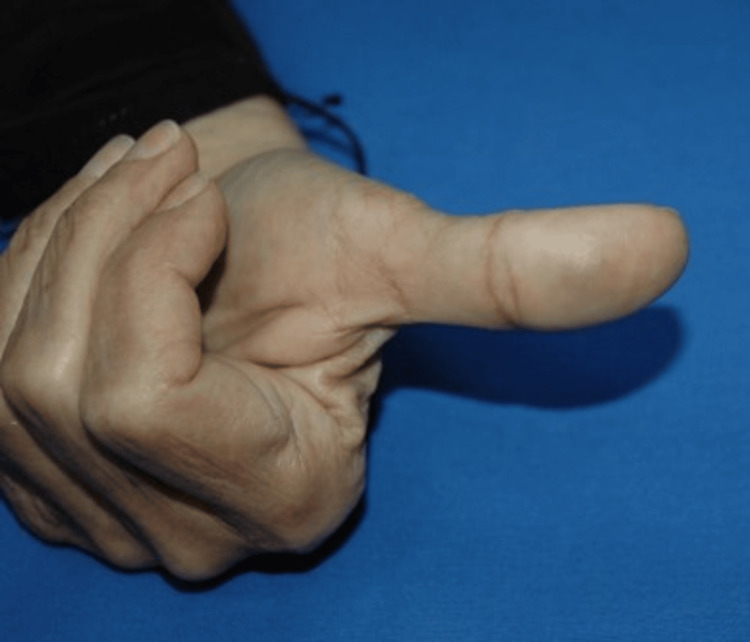
The lesion on the patient’s left thumb

**Figure 2:**
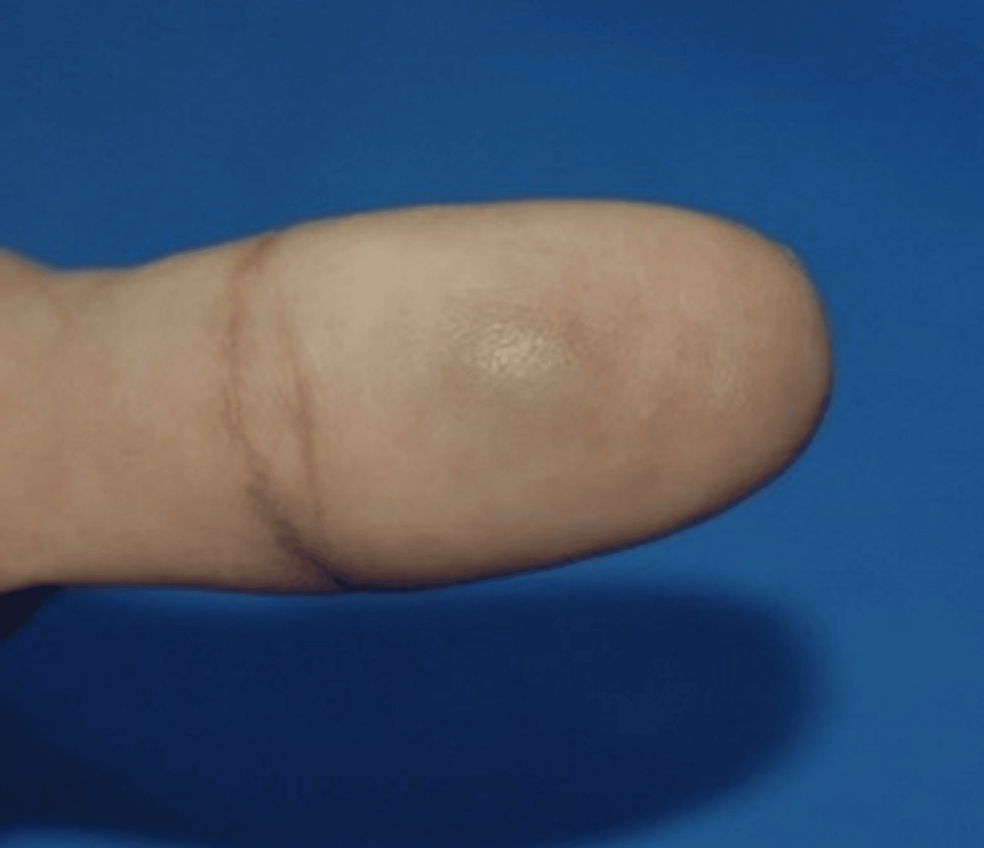
This is a close-up image of the lesion

On physical examination, there was a 2-mm, firm, bluish nodule on the distal palmar aspect of the left thumb, which was tender on palpation. There were no bruise-like patches in the overlying or adjoining skin. The surface of the lesion was smooth with no changes in the surrounding skin (Figures 1, 2). The Tinel sign was negative, and there was no paresthesia across the distribution of the affected neighboring digital nerve. There were no other similar lesions on the patient’s right and left hands.

**Figure 3:**
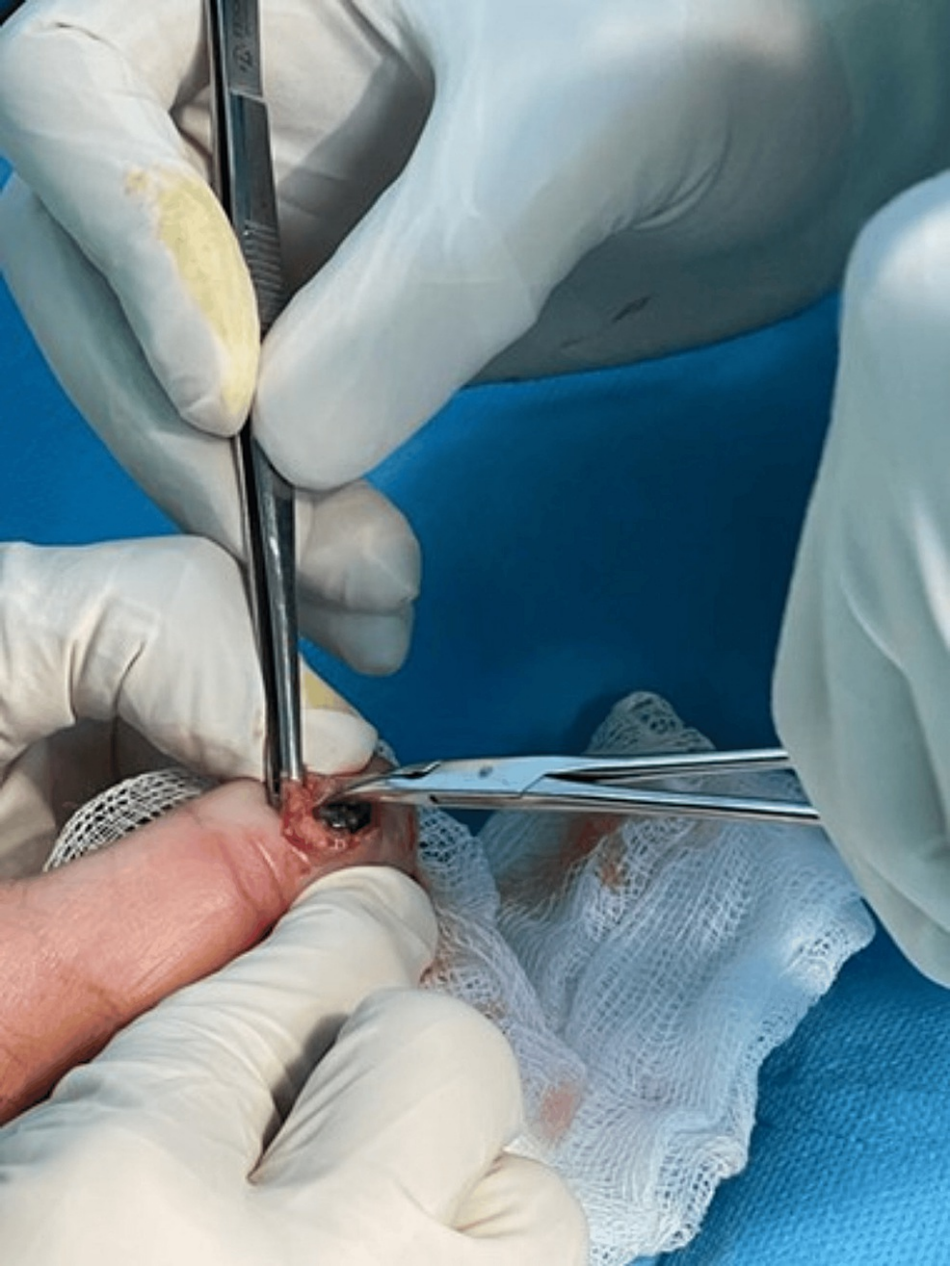
Intraoperative image of the lesion

The lesion was excised under local anesthesia (Figure 3).

**Figure 4:**
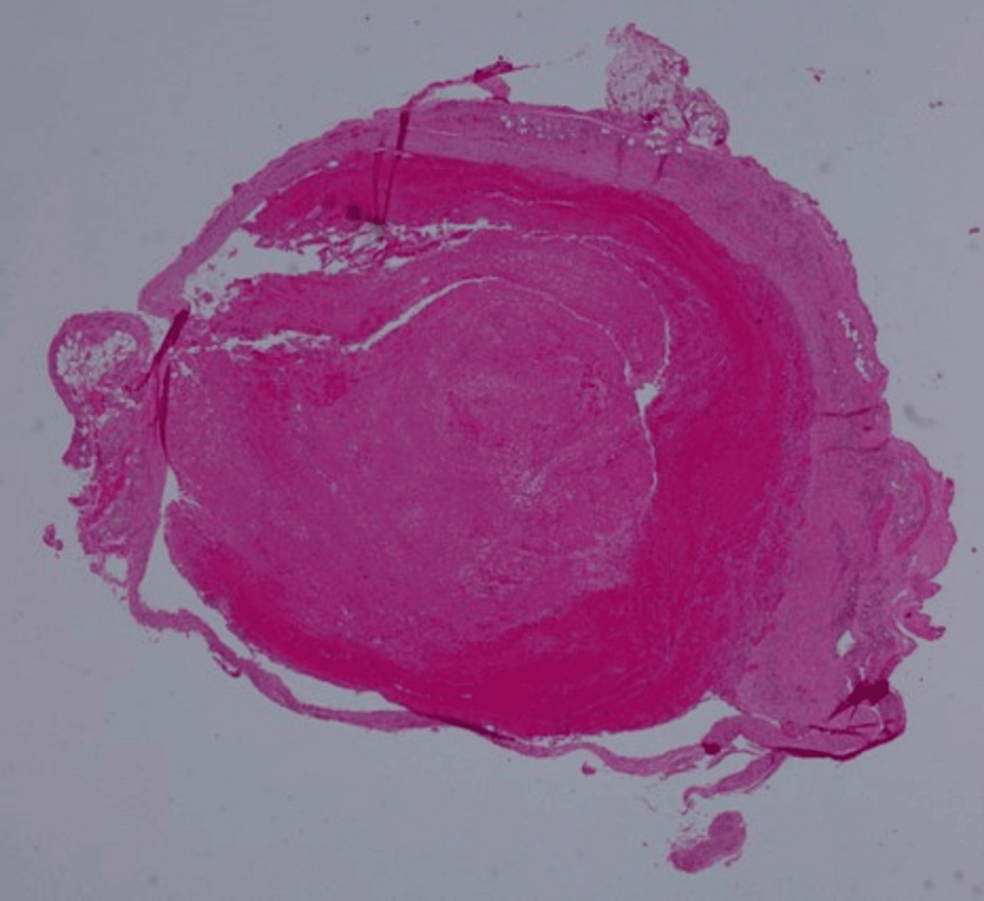
The vascular lesion showing intraluminal organizing thrombus (H&E, x20)

**Figure 5:**
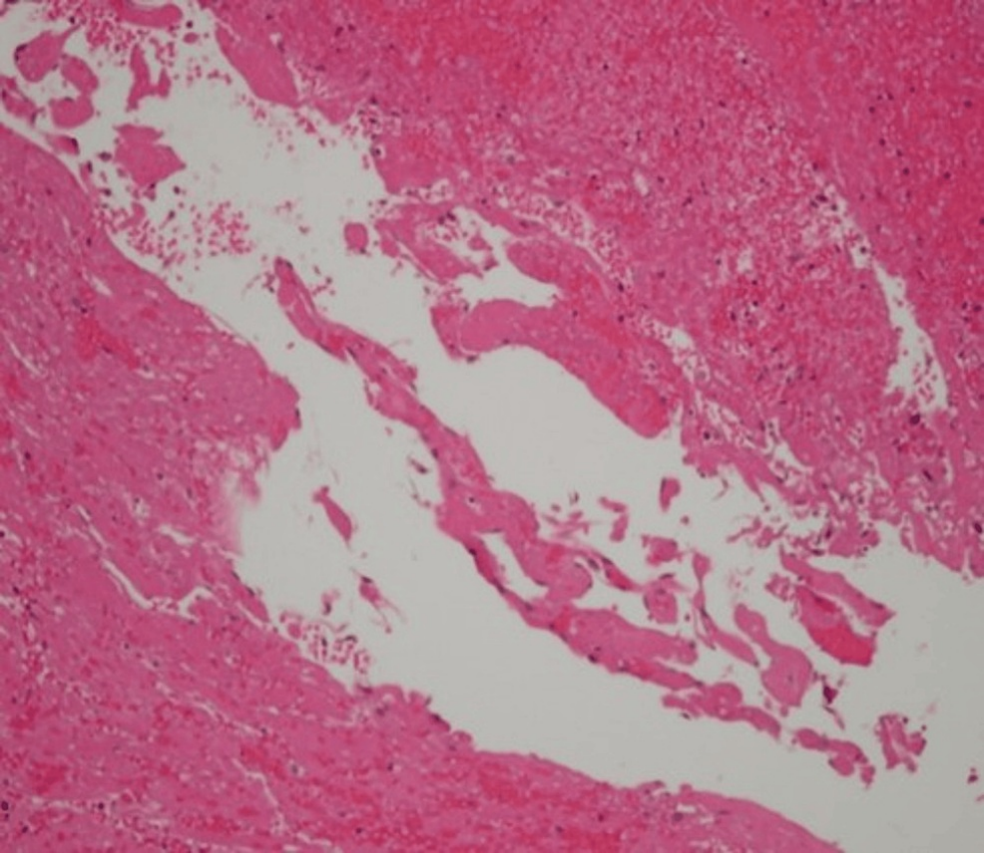
A focal intravascular papillary proliferation of plump endothelial cells with hyaline core (H&E, x40)

**Figure 6:**
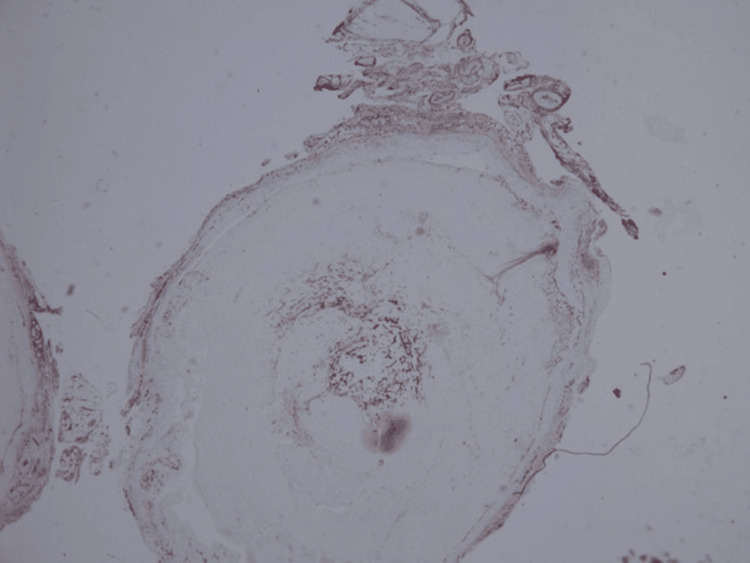
Endothelial cells positive for CD31 (x20) CD31: cluster of differentiation 31

The excised lesion was compassed, bluish, and rubber-like. The specimen was bisected and processed in one cassette (Figure 3). Histopathological examination (Figures 4, 5) revealed dilated vascular spaces with clotted blood and papillary endothelial proliferation inside the blood vessel’s lumen. Papillae, composed of a single layer of endothelial cells with collagenized core were present. Anastomosing vascular channels lined with bland and plump endothelial cells were also found; the endothelial cells were positive for CD31 (Figure 6). The histological diagnosis was consistent with IPEH (Masson’s tumor).

**Figure 7:**
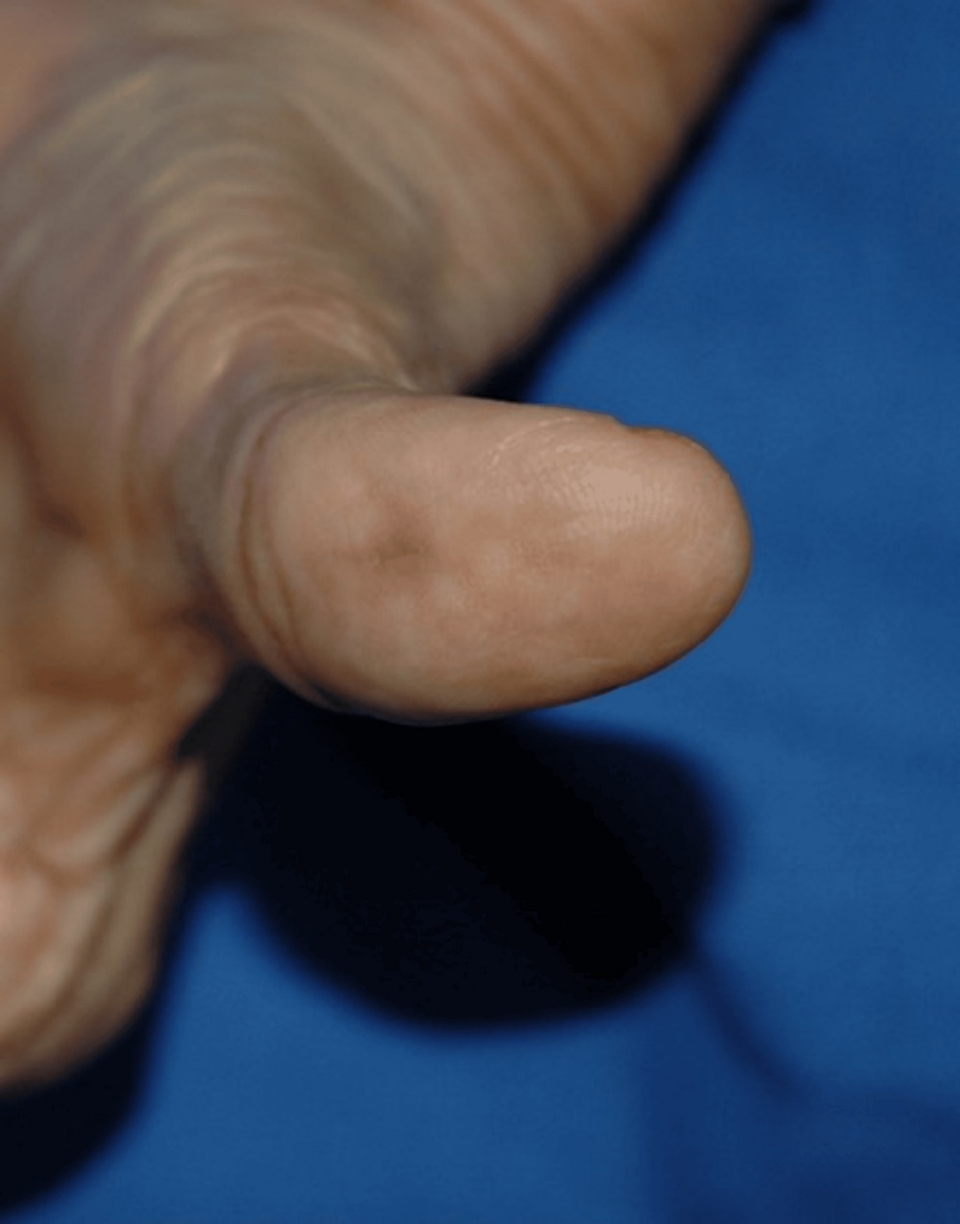
The wound three months following excision

Subsequent follow-up of the patient showed that the area of excision healed with minimal post-inflammatory pigmentation (Figure 7). The patient expressed her satisfaction with the outcome of the excisional surgical procedure.

## Discussion

Papillary endothelial hyperplasia remains an uncommon benign vascular lesion that can occur in any vessel in the body. Approximately 2% of vascular tumors of the skin and soft tissues are caused by IPEH [9]. The pathophysiology of IPEH is not well understood, but the majority of current research suggests that it is caused by endothelial cells proliferating in blood vessels in response to post-traumatic thrombus formation, which is also linked to the organization and recanalization of thrombi. The order in which the disease advances is still debatable though; the primary process is either endothelial proliferation with subsequent thrombus development or the thrombus appears first and forges papillary proliferation [10].

The three differentiable IPEH subtypes are: (1) the primary type (56%) originates in dilated vascular spaces and presents with a small subcutaneous mass, frequently in fingers; (2) the secondary type (40%) develops in an antecedent vascular lesion like pyogenic granulomas, hemangiomas, or vascular malformations; and (3) the extravascular type (4%) evolves from a pre-existing hematoma, and these lesions are larger than the other-type lesions [10]. In our case, the lesion developed within a dilated vascular space, which is consistent with the primary subtype of IPEH.

The lesion frequently presents in the third and fourth decades of life with a predilection to females [10]. It often manifests as a small subcutaneous lump that may or may not be painful; the overlying skin may also become blue or reddish in color [10]. However, our patient was in her seventh decade and her lesion was a painful bluish nodule.

Trauma frequently antecedes the development of this tumor; however, a clear history of trauma is elicitable in only 4% of the patients [11]. Any history of significant trauma was not forthcoming from our patient.

It is difficult to diagnose IPEH clinically, as there are no specific clinical findings, hence histopathological examination is the gold standard for diagnosing IPEH [12]. A significant histopathological feature of IPEH is the appearance of hyperplastic endothelial cells covering papillary structures, intravascularly. A close association with the organizing thrombus contributes to the diagnosis [10]. Our case showed features consistent with these. Ultrasound may help in differentiating IPEH from other soft tissue masses by detecting one or more vessels associated with the lesion [12].

Other benign and malignant vascular tumors, such as glomus tumor, glomerulovenous hemangioma, venous lake, blue nevus, neurogenic tumor, and angiosarcoma, should be distinguished from IPEH [10]. Despite IPEH's benign nature, it is important to have an accurate histopathological diagnosis based on its similarity with angiosarcoma, a tumor of the blood vessels. Angiosarcomas are usually not present within the blood vessel lumen, which is a key differentiating histopathological feature between angiosarcoma and IPEH. Moreover, IPEH endothelial cells are not necrotic but have prominent pleomorphism or major mitotic activity and uniformly formed sheets as opposed to angiosarcoma [10].

As IPEH does not advance to a malignant neoplasm and is not reported to recur after diligent surgical excision, the preferred treatment, with a favorable prognosis, is surgical excision [1,13]. The case that we are presenting here was treated with total surgical excision and there was no recurrence after six months of follow-up.

## Conclusions

Masson’s tumor or IPEH is an entity difficult to diagnose clinically. We report here an adult Kuwaiti female with a mass in the thumb histologically proven as IPEH, which was resected. There are no identifiable specific features to diagnose IPEH clinically. Therefore, histopathological examination is the gold standard for diagnosing IPEH. Also, a helpful aid in these cases could be a pre-operative ultrasound examination that may differentiate IPEH from other soft tissue masses. The message we would like to convey is that IPEH, a rare benign tumor composed of reactive endothelial cells and a thrombus within a vascular space, should be kept in mind when encountering a painful bluish skin lesion. Since Masson’s tumor can mimic other skin lesions clinically, histopathology is a definitive technique for diagnosing IPEH. Surgical excision is the treatment of choice, and the prognosis is excellent with no recurrence if the excision is comprehensive.
